# Diabetes management interventions for homeless adults: a systematic review

**DOI:** 10.1007/s00038-020-01513-0

**Published:** 2020-10-23

**Authors:** Janice Constance, Joanne M. Lusher

**Affiliations:** 1 School of Psychology, London Metropolitan University, London, UK; 2 Institute for Research in Healthcare Policy & Practice, University of the West of Scotland, London Campus, 2 Clove Crescent, 7th Floor, London, E14 2BE UK

**Keywords:** Type 2 diabetes, Systematic review, Diabetes management

## Abstract

**Objectives:**

Recent studies investigating diabetes show that inequalities to access appropriate care still persists. Whilst most of the general population are able to access a suitable quality of care, there are a number of groups who fail to receive the same standard. The objective of this review was to identify existing diabetes management interventions for homeless adults.

**Methods:**

A literature search was conducted in February 2017, and repeated in September 2020.

**Results:**

Of the 223 potentially relevant articles identified, only 26 were retrieved for detailed evaluation, and 6 met the inclusion criteria. Papers focusing on the management of diabetes in homeless people were included. The studies used interventions including diabetes education; medication support and supplies for blood monitoring; improvements in self-care behaviours; improvements in diabetes control; patient empowerment/engagement; and community engagement/partnerships.

**Conclusions:**

Effective strategies for addressing the challenges and obstacles that the homeless population face, requires innovative, multi-sectored, flexible and well-coordinated models of care. Without appropriate support, these groups of people are prone to experience poor control of their diabetes; resulting in an increased risk of developing major health complications.

## Introduction

In recent years, there have been numerous developments in the treatment of diabetes (Marín-Peñalver et al. 2016). Better insights into the pathogenesis of diabetes, the availability of new therapies, the importance of structured care and risk factor control have all contributed to the transformation in which most people receive diabetes care (American Diabetes Association 2011). It has been noted that people with diabetes should have a dominant role in their own diabetes care and treatment, to effectively implement any necessary lifestyle changes to their diet, medication management, exercise regime, smoking status and attendance to their medical appointments, such as blood glucose testing and inspections of the eyes and feet (Heinrich et al. 2010). However, inequalities in access to health still persists, whilst the vast majority are able to access an appropriate quality of diabetes care, there are still groups of people that are difficult to reach and therefore fail to receive the same standard of care (i.e. people who are classified as homeless) (Diabetes UK 2016). These groups of people are suggested to be on the outer edge of the healthcare system, do not have regular contact or interactions with healthcare professionals and frequently report with major complications or as emergency cases. Healthcare professionals’ ability to provide a high quality of care to these groups of people is further affected due to the scarcity of research and the lack of understanding of this particular population group needs and complications (Bellary 2011).

The United Nations global survey conducted in 2005, estimated that 100 million people were homeless worldwide and as many as 1.6 billion people did not have access to appropriate accommodation (Habitat 2017). The most recent UK government figures suggest that there are over 14,930 individuals classified as homeless and a further 74,630 households in temporary accommodation in England alone. (National Statistics 2016). In comparison, on average it is estimated that 553,742 people in the United States will experience homelessness at any given point (US Department of Housing and Urban development 2017). Whilst it is estimated that more than 235,000 Canadians experience homelessness in a year (Gaetz et al. 2014). The definition of homeless is quite broad, as many households have more than one individual living at an address, the actual number of homeless individuals could be considerably larger than estimated. There is limited information regarding the prevalence rates of diabetes amongst the homeless population in the UK. However, diabetes prevalence rates have been reported in those living in France, with rates of 6.2% in the homeless as compared with 4.9% in the general population (Arnaud et al. 2009). Reports from Canada suggests a prevalence rate of about 3%, which is not as prominent compared to that of the general population (Hwang and Bugeja 2000).

Obstacles in diabetes self-management are more prominent in homeless population due to the difficulties in relation to the social determinants of health, including: lack of family and social support, unemployment, mental illness, food and shelter instabilities (Baggett et al. 2010). All these factors can affect an individual’s capacity and ability to adhere to the management of their diabetes care (i.e. management of their diet, medications, and self-monitoring of their blood glucose) (Hwang and Chiu 2006). Hwang and Bugeja (2000), found that one of the major difficulties reported by homeless people were difficulties in prioritising their diabetes conditions over other problems they may be experiencing, accessing and securing insulin needles and syringes, obtaining medications and exercising.

Homeless people are suggested to be one of the hardest to reach groups, due to the constant change in their abode, accessing health care might not be as simple compared to the general population (Jones and Gable 2014). Even though classified as vulnerable, there is very little research into this population group and information about recognising and providing appropriate treatment to these individuals remains poor. More research is needed to identify effective strategies to improve care. Few studies have suggested that addressing this particular population’s health care needs requires a coordinated effort from healthcare professionals and other organisations to ensure better access to community and specialist diabetes services (Gilani 2014; Jones and Gable 2014). Developing registers and contacting these individuals through social networks and charities could be explored further. Sharing of best practice and developing innovative approaches may be another way to highlight the issues related to the homeless population.

To the best of our knowledge, there is no specific systematic review investigating the efficacy of diabetes management interventions in homeless adults. For this review, the term homeless was defined in accordance with the UK Homelessness legislation, first introduced in 1977 as the housing homeless persons Act (Burrows et al. 1997). The legislation defines an individual as homeless if they don’t have the legal right to reside in an accommodation, or if it is not suitable for them to live in.

To identify diabetes management interventions for homeless adults, the specific questions that were addressed by this review were:

- What are the existing interventions for managing diabetes amongst homeless adults?
- What are the principles and barriers to successful management of diabetes in homeless adults with this disease?

## Method

This review was initially registered with PROSPERO in 2017 (record CRD42017070144).

### Eligibility criteria

The Table 1 represents the inclusion and exclusion criteria for papers used for this review.

**Table 1:** The inclusion and exclusion criteria used during the screening process

*Inclusion criteria*	
Types of studies	Studies investigating the management of diabetes amongst homeless adults
	Studies written in English
	Studies conducted in any year
	Full-length studies published in peer review journals
	Primary studies, using either retrospective or prospective design or either quantitative and/or qualitative design (studies with measurable outcomes); including clinical trials quasi experimental studies
Types of interventions	Studies were included if the outcomes were measured for the diabetes management intervention and consisted of adults who has Type 2 diabetes (age ≥ 16 years)
Types of outcome measures	Studies were included if the intervention outcomes included one or more of the following: Glycaemic control: HbA1c, blood glucose levels Cardiovascular risk factors (e.g. Cholesterol, blood pressure, weight, BMI and serum creatinine Mortality Hospital admissions
	Studies were also included if the intervention outcomes included self-reported measures such as: Diet improvement Patient satisfaction Well-being, quality of life, perceived health scores on a validated generic or disease specific measure Medication adherence
*Exclusion criteria*	
Types of studies	Non English language
	If it was a commentary, editorial or case study on transition
	The primary focus was not the management of diabetes among homeless adults
Types of interventions	Participants without a diagnosis of Type 2 diabetes
	Participants who are not categorised as homeless
	Participants younger than 16 years of age, which included trials that involved both children and adults
	Specifically targeted healthcare professionals
	Focused specifically on the management of being homeless
Types of outcome measures	Focused specifically on the prevalence of diabetes
	Focused solely on the clinical improvements as the only outcome measure (because management interventions can also be targeted towards behaviour change, which does not always lead to clinical improvements)

There were no restrictions on variables such as culture, stage of illness, occupational class or education. The searches were limited to the English language as the time and cost of translation were not feasible within this reviews timeline.

### Search strategy

This involved methodically reviewing the Cochrane Database of Systematic reviews, PsychINFO, PubMed and Web of Science databases. All database searches were carried out in February 2017, and repeated in September 2020, using the following terms in combination with Boolean operators: Diabetes mellitus; Diabetes; Diabetes mellitus type 2; Hyperglycaemia; Hypoglycaemia; non-insulin dependent diabetes mellitus; glucose intolerance; #1 OR #2 OR #3 OR #4 OR #5 OR #6 OR #7 OR #8; Homeless person; Homeless; Homelessness; Street people; Unstable house; #9 OR #10 OR #11 OR #12 OR #13; #8 AND #14; Health Education OR Client Education OR Health Literacy; Coping Behaviour OR Self Care Skills OR Disease Management; Treatment Compliance OR Health Attitudes; Health Behaviour OR Health Promotion; Intervention; Program Evaluation; Behaviour Therapy; Lifestyle; #16 OR #17 OR #18 OR #19 OR #20 OR #21 OR #22 OR #23; #8 AND #14 AND #24. In addition to this search strategy, a manual search of reference list of all relevant papers identified was also conducted.

### Study selection

All the titles and abstracts of the potentially relevant papers were examined by both authors to determine whether the paper met the inclusion criteria set. The full text of included titles were then accessed to determine eligibility by both reviewers. Papers that met the inclusion criteria at this stage were included in the final analysis.

### Data extraction and study quality

A standardised data extraction form was developed (see Table 2). Data was extracted using the following variables: Study characteristics (i.e. name of primary author, publication year, country of study, and the research aims and objectives); Participant characteristics (i.e. age, gender and other sociodemographic data); Study design; descriptions of interventions; intervention measures and the key findings. The quality of each of the papers identified was assessed using the Downs and Black checklist (Downs and Black 1998). The checklist has been found to have a high internal consistency, inter-rater reliability and test–retest reliability (To et al. 2016). The Downs and Black checklists assesses papers on items relating to reporting. For example objectives, participants, the outcomes, study findings, cofounders, internal validity, external validity and power. A score of 31 is the maximum that could be gained using this checklist (Downs and Black 1998). Discrepancies were resolved by two reviewers. It was concluded that a meta-analysis would not be an appropriate method of evaluating the findings as the included studies differed in the study aim and outcome measures. In addition the quality assessment revealed the majority, five out of six studies were of low quality. Therefore there would have been no meaningful outcome in pooling together the data.

**Table 2:** Summary of studies included in this review

Authors (year)	Research aims	Sample size (%male) %Homeless	Mean age (range)	Setting	Description of intervention	Assessment of intervention	Key results	Quality score^1^
Thompson et al. (2014)	Assess the effectiveness of the GMV program for individuals with a diagnosis of diabetes or at risk of developing diabetes	52 participants in GMV program. 9 took part in interviews (100%) 100%	54.5 years (46–62)	Community health Care in Canada	The intervention consisted of monthly GMV’s. Each session included group discussion of a topic relating to diabetes management, medical care, an evaluation of behavioural goals	Semi-structured interviews	Participants described how the group medical visits programme was implemented, their thoughts on the qualities of a good facilitator. Also what the role of the group members had in supporting their behaviour change in diabetes management and provided feedback and suggestions for improvement	14
Davachi and Ferrari (2012)	To identify the barriers that could impact the homeless participants’ ability to manage their diabetes To develop an accessible and effective diabetes management support for the homeless population especially those at risk or already diagnosed with diabetes	524 (74%) 100%	Not recorded (≥ 18 years)	Calgary Drop-in & Rehab Centre (CDIRC) in Canada	The project for the management support included components such as diabetes awareness, screening, group visits and also individual case management	A reduction in participants fasting blood glucose (FBG) and their HbA1c levels. Which will be measured at 3 and 12 months	Amongst the 524 participants’ screened, 11% were found to have pre-existing diabetes, 16% had high blood glucose levels Baseline results and results captured at the 3 to 12 months follow-ups were only available for 10 patients with pre-existing diabetes. However although the low numbers of follow-up data collected, the mean reductions in FBG of 4 mmol/L and HbA1c of 1.1% is significant for this population	21
Pauley et al. (2016)	To evaluate the feasibility of cluster care and a supportive housing model integrated for the participants	212 (35%) 100% Participants with Diabetes (8%)	55.5 years (37–76)	Three participating supportive inner-city housing facilities in Toronto, Canada	Integration of cluster care and supportive housing models. In the supportive housing model, services and housing are combined in the same location Components of cluster care included the following: (1) care at a given site is provided by a single provider agency. (2) The agency deploys teams of care providers instead of individual workers. (3) Care plans are structured on the basis of assessment and care plan specific tasks rather than block of time	Goal attainment scale and interviews	During a 15 month period, 20 clients received this service (pre-implementation). Which increased to 147 clients (post-implementation) during a 16 month period, with a 60% reduction in cost The results shows that regular team meetings promoted efficient service delivery; greater client satisfaction associated with goal achievement and finally reported client satisfaction where staff and client goals were aligned closely together	17
Beggs and Karst (2016)	To assess the effectiveness of an education programme led by pharmacy students with adults experiencing homelessness	17 pharmacy students (NR) 0% Participants with diabetes 8 (100%) 100%	NR	Local outreach organisation in Nashville, USA	Bingo games focused on a wide variety of questions related to the health topic, including basics of the disease, anatomy, statistics, medicines, diet, lifestyle, environmental concerns, and common misconceptions. Each group led a one-hour class using Bingo games over one week	Survey	In total 37 surveys were completed. The results showed that the classes led by pharmacy students were effective in increasing the knowledge of each of the health topics presented. All participants stated they would attend future classes that are led by pharmacy students	15
O’Toole et al. (2010)	To determine if a population tailored approach delivered to homeless veterans of how primary care is organised would lead to better health care and outcomes	Homeless: 79 (96%) 100% Control: 98 (96.7%) 100%	Homeless: 51.8 SD = 0.94 (NR) Control: 52.9 SD = 7.7 (NR)	The participants in the intervention group voluntarily enrolled in a homeless oriented primary care clinic, located in Providence VA Medical Centre (USA) Participants within the control group were new to primary care	The Homeless- Oriented Primary Care Clinic was structured to address 4 core elements of the chronic care model specifically tailored to homeless persons. The clinic had an open-access care model with onsite services that included food, housing assistance, clothes and veterans’ benefits	Clinical assessment measures such as blood pressure checks for hypertension, HbA1c for diabetes, and low density lipoprotein [LDL]	The patients showed improvements in their hypertension, diabetes and in lipid control. The use of the primary care was higher during the initial 6 months but started to stabilise 6 months after The use of the emergency department also saw an increase, although there was a 40% decrease in the non-acute emergency department visits Excluding the abuse of substances and admissions due to mental health, hospitalisations decreased amongst the homeless veterans in the 2 6 months periods compared to the control group	26
Savage et al. (2014)	To examine the rate of retention in homeless adults and investigating the feasibility of a CDSM diabetes intervention	9 (NR) 100%	NR	The participants were recruited through a nurse-led clinic for homeless people in Ohio (USA)	The intervention included the use of educational sessions with the integration of behaviour change strategies. In addition the intervention also included the use of assessments led by nurses and outcomes achievements	Surveys were conducted at baseline and also at 12 weeks Participants’ self-efficacy was measured using the Managing chronic Disease questionnaire Health behaviours was measured using a cognitive symptom management questionnaire and a communication with physicians’ questionnaire	Only 5 out of the 9 participants stayed for the full 12 weeks, whilst 2 out of 3 participants completed the intervention	16

CDSM = Chronic Disease Self-Management; FBG = Fasting Blood Glucose; GMV = Group Medical Visits; SD = standard deviation; NR = not reported

^1^ Assessed by Downs and Black checklist

## Results

**Fig. 1:**
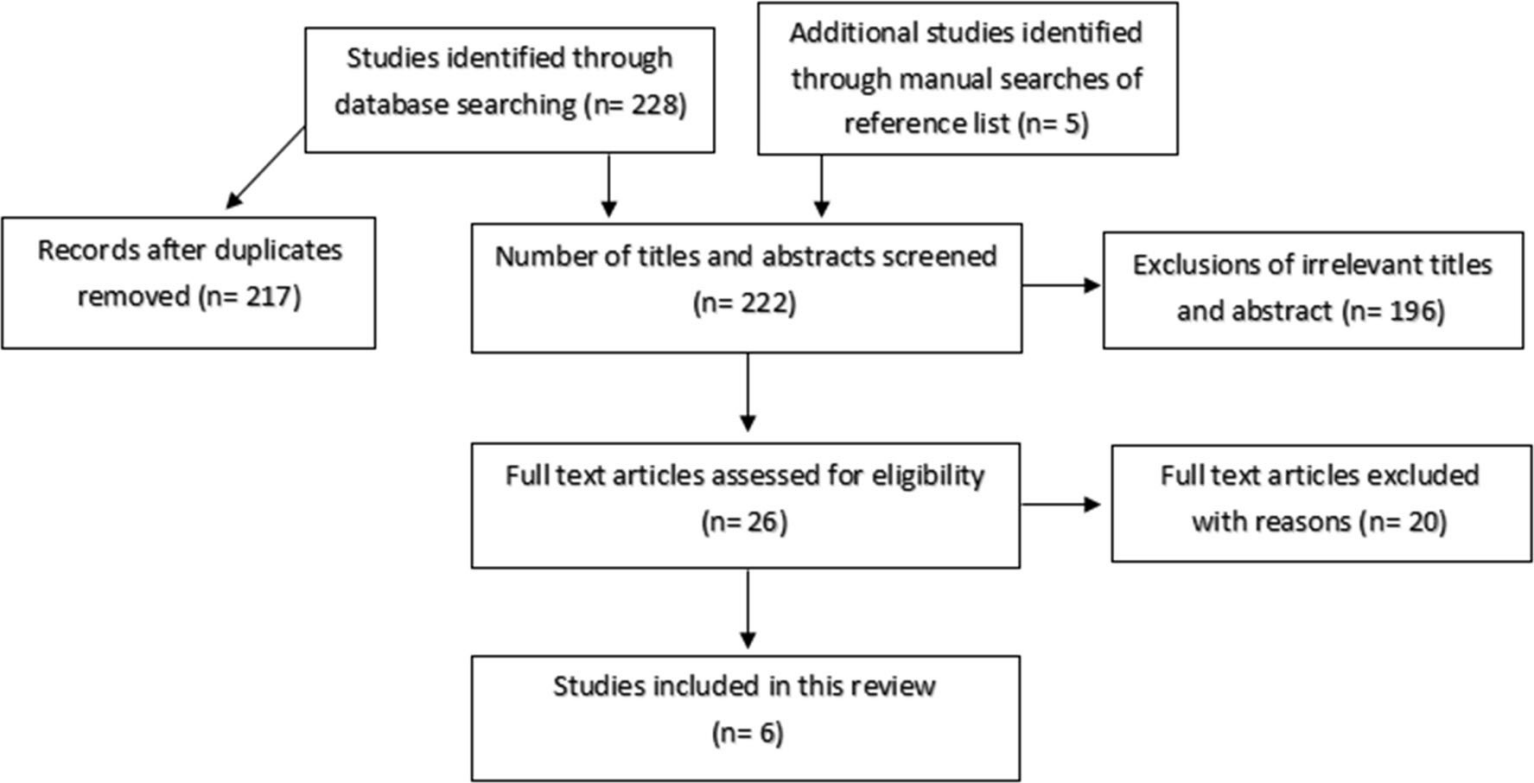
Flowchart of selection process

The search strategy generated a number of potentially eligible papers. After screening through titles and abstracts, 26 papers were initially identified as potentially relevant and their full texts were accessed to determine whether they met the inclusion criteria. Of those 26 papers, 20 were excluded as their participants did not include homeless adults, or did not include any interventions for the management of diabetes as shown in Fig. 1. After carrying out a second search, no further studies met our criteria for inclusion. Therefore, a total of six papers were included in the analysis of this review (characteristics of included studies are presented in Table 2).

All of the studies found were conducted in developed countries, three of the studies included within this review were conducted in Canada (studies 1, 2 and 3) and the other three studies were conducted within the United States (studies 4, 5 and 6). All of the studies included participants who attended organisations that are dedicated in serving the vulnerable populations. One study included patients from a nurse-run clinic (study 6). Another study recruited participants from three supportive inner-city housing facilities (study 3). One study recruited participants who volunteered at a homeless oriented primary care clinic (study 5) and another study included participants from a rehab centre (study 2). One of the study recruited participants from a community health care (study 1) and lastly one study recruited participants from a local outreach organisation (study 4).

The studies included in this review had an average number of 167 participants (range 8–524). Across all six studies the mean age of the participants was ranged from 37 to 76 years old. All of the studies recruited a majority of male participants which ranged from 35 to 100% of the participants. The definitions of homeless also varied across all the studies. However, two studies included the definition of homelessness as a criteria of their study enrolment (studies 5 and 6). While one study recorded the duration of homelessness (study 3), no details were provided about the participants housing transitions. Two of the studies included previous diabetes diagnosis as a criteria for study enrolment (studies 1 and 6). Information on the participants’ ethnic background was included in half of the studies (studies 1, 4 and 5). Most of the participants in the studies were individuals from minority populations, for example African-American. However, no studies investigated whether there was a difference in outcomes by ethnic race. Out of the six studies, only one study provided details on their participants’ education level, employment status and income (study 4).

### Interventions that exist for managing diabetes in homeless adults

As can be seen in Table 2, the majority of the studies measured the effects of an intervention for diabetes care, which included a participant questionnaire alongside various assessments for diabetes. Two of the studies (33%) involved a medical assessment as one of the outcome measure of their intervention (studies 2 and 5). One study retrieved information about participants’ diabetes management through self-reported survey data only (study 6). Half of the studies assessed the effectiveness of their intervention through gaining feedback from the participants (studies 1, 3 and 4). None of the studies assessed participants’ diabetes management over a long period of time.

The methodological quality reported in most of the studies were generally moderate with a median score of 18 (range 14–26). The main study objectives were clear in all 6 of the studies, and the main outcomes were adequately described. Two of studies lacked participants’ characteristics such as age, gender, other sociodemographic data and how participants were recruited (studies 3 and 4). Details on participants who lost a follow-up were not reported in any of the six studies. Majority of the studies scores were low on internal and external validity (studies 1, 3 and 4). Whilst one study was determined to be insufficiently powered in order to detect clinically meaningful differences (study 6).

### Principles and barriers to the successful management of diabetes in homeless adults

All studies included within this review highlighted not only the need of diabetes management programmes for the homeless but also the barriers and obstacles that this population group faces in accessing care for their diabetes. There is a need for effective and innovative models of care to help overcome these disparities. A few studies have suggested that diabetes is a “holistic and social” disease amongst people who are homeless. It is described as an additional challenge to a person’s daily life struggles (studies 2, 3 and 5). Effective strategies for addressing the challenges and obstacles that the homeless population face demands for not only well-coordinated models of care, but also for them to be flexible, diverse and most importantly multi-sectored. All individuals with diabetes needs to be understood and consulted as a “whole person”. This acknowledgement will build rapport between patients and healthcare professionals, and ultimately improve their care (studies 1, 2 and 3). Two of these studies suggested that healthcare professionals should be knowledgeable about the process of behaviour change, understand how social disadvantages might influence the change process and furthermore should be able to provide appropriate referrals, facilitate discussion and mobilise professional support to address the challenges that this particular population face (studies 1 and 3). It is necessary that healthcare professionals receive sufficient and appropriate training to understand how to incorporate the principles of patient-centred care when working with the homeless population. A collaborative relationship between the healthcare professionals and the patients will likely lead to both greater concordance and goal achievement within the management of the patient’s diabetes. Diabetes management interventions reported in the studies identified by this review were categorised as:

### Diabetes education

Majority of the studies included in this review provided participants with educational sessions on what diabetes is, educational materials and access to disease management classes as part of the described intervention provided (studies 3, 4, 5 and 6). However, in studies 3, 4 and 6 there were no outcomes related data to enable the comparison of the effects of participants with or without diabetes following a class (such as improvements in HbA1c levels for participants with diabetes). It is unclear in study 5 whether the recorded decrease in HbA1c was a direct result of participants attending the diabetes education component of the intervention.

### Medication support and supplies for blood monitoring

The most common challenges that are experienced by the homeless population includes the lack of access they have to medication such as insulin due to not having health insurance and the lack of support in gaining prescriptions. A few studies included services whereby participants received blood glucose monitoring supplies (studies 2 and 6), medication, advice on medication management and assistance with their prescriptions (studies 2, 3 and 6). However there was a lack of information across all 3 studies on the effect the supplies had on the management of diabetes.

### Improvements in self-care behaviours

Participants also received dietary supplements, had access to food sources and assistance with meal preparations in the majority of the studies (studies 2, 3, 5 and 6). However, only one study noted that there was an improvement in the access to healthier foods by participants (study 2). Although baseline and follow-up assessments were done for all the participants who had diabetes in this study, only 15 participants (34%) were included in the final assessments. It was noted that there was an improvement in access to healthier foods, for example: fruits, vegetables and whole grains. A dietitian was also available to support the participants in making the best choices from the healthy food options available. However the study found that only 27% of the participants were consuming 3 meals a day, this is because the majority of the participants left the shelter after having breakfast. In one study participants were also provided with sessions on preventing complications whilst living on the streets which included an introduction to physical activity, stress management and relaxation strategies (study 6).

### Improvements in diabetes control

Amongst the six studies included in this review only two studies recorded improvements in the participants’ blood pressure, LDL and HbA1c levels (studies 2 and 5). One study found that amongst their entire sample size only 28 of the participants with diabetes had their baseline results available. Amongst these participants, 16 (75%) had elevated fasting blood glucose (mean 9.5 mmol/L; min 5.0 mmol/L, max 23.4 mmol/L). However both the baseline and follow-up results were only recorded for 10 (36%) of these participants. The 3 and 12 month follow-up results showed that there was significant improvements in their fasting blood glucose and HbA1c levels with a reduction of − 4.0 mmol/L and − 1.1% respectively (study 2). Whilst another study found that there was a decrease in HbA1c levels (− 2.3%) within the intervention group and contrastingly an increase within the control group (HbA1c: + 0.2%). In study 5, 65.4% of the participants within the intervention group achieved their target goal in comparison to 45.5% in the control group. The study also noted that there was a decrease in the LDL levels in both the intervention and control groups (− 6.4 mg/dL and − 1.1 mg/dL respectively).

### Patient empowerment and engagement

As the homeless population has a daily struggle in securing the basic necessities such as food and shelter, diabetes in this population often goes unnoticed or not appropriately recognised because their symptoms are screened and diagnosed as other diseases or conditions. One study however, was successful in raising awareness on diabetes and empowering their participants to manage the disease (study 2). Whereas another study found that participants perceived group peer support as enhancing their capacities for diabetes management through group problem solving, modelling, the provision of information, emotional support, and social comparison (study 1). Supportive intragroup relationships have long been recognised as a therapeutic mechanism in group therapy, and are increasingly seen as a motivational tool in group-based diabetes self-management programming.

### Community engagement and partnerships

Two studies out of the six included in this review concluded that having a multi-sectored approach results in greater community support and actions with aiding the homeless. There is a need for further partnerships with other organisations such as food agencies and pharmaceutical companies which would prompt the provision of medications, food supplements and blood glucose monitoring supplies (studies 2 and 5). These two studies (study 2 and 5) also included on-site integration of homeless-specific services within their interventions (i.e. housing and benefits assistance staff available on-site). One of these studies interpreted the improvement in blood pressures, HbA1c readings, and LDL values as a direct result of the participants having an increased contact with primary care and management services (study 5). Whereas two other studies (studies 1 and 6) concluded that healthcare providers play an important role in fostering supportive and helpful relationships among group members by orienting participants to their roles in the group, monitoring and encouraging supportive interactions among group members, and modelling positive regard.

## Discussion

The aim of this review was to identify diabetes management interventions specifically designed for homeless adults. Homelessness is a major social problem worldwide, and difficult to quantify due to a lack of a universal definition of homelessness. In developed countries, data is collected based on household reports, meaning reports do not take into account the hidden homeless i.e., those living temporarily with friends or family (Busch-Geertsema 2010). Being homeless comes with a set of comorbidities and challenging social problems, which can often be overwhelming for the individuals or the healthcare teams. Using a patient-centred approach is key to working with this particular client group. As a healthcare professional providing specialist diabetes care, it may be a requirement to help individuals overcome barriers and help them navigate what can be a confusing array of services. Using resources such as the mental health service and the third sector can help people overcome their barriers to achieving better diabetes care.

Even though it is suggested that people who are homeless have poor control over their diabetes, it is difficult to establish this without having robust data. The reported differences in glycaemic control and the rates of complications between studies may reflect the differences in population backgrounds of homeless people and methods of data collection. Higher rates of foot complications have been reported in one study, but this has not been shown in other studies (Arnaud et al. 2009). While there has been little empirical data published regarding interventions for homeless people with Type 2 diabetes, studies have emphasised the daily struggles and obstacles that the homeless population face to meet their basic needs (i.e. finding shelter and food) were more of a priority to them than effectively managing their diabetes. Addressing these obstacles that the homeless population face in accessing diabetes care is a challenge as they are often hard to reach or need to be approached in different settings. Other problems, such as mental health problems and substance abuse, are also common in homeless people and may also contribute to poor diabetes outcomes (Hwang and Bugeja 2000; Hwang et al. 2011).

Homeless people living in shelters are less likely to adhere to meal times or even have access to healthy food items. They are also less likely to monitor their blood glucose levels or see a doctor (Kalinowski et al. 2013). Some of the other problems reported by homeless people with diabetes include difficulties in exercising, scheduling and prioritising diabetes over other problems, securing insulin needles and other medications (Hwang and Bugeja 2000).

Although there are limited number of strategies in place to aid the improvement of diabetes care that the homeless population receive, studies that have shown that community based and disease management models targeted specifically at diabetes are effective within this population (Plumb et al. 1996). Community based services that are nurse-led and supported by various multidisciplinary teams are found to be effective in addressing the socioeconomic barriers vulnerable populations encounter when accessing appropriate care (Savage et al. 2008). Results also confirm the importance of engaging vulnerable populations alongside the healthcare system and key partners within the community to address the obstacles in improving health outcomes. Further work needs to address the significant gap that exists in delivering appropriate diabetes care, i.e. services that are not only accessible but sustainable to the homeless population. Such knowledge is vital when it comes to planning and delivering service models that are effective in improving health. Healthcare professionals that provide care and services to this population group also face complex challenges, for example adapting their practice in order to appropriately address the inflexibilities of diabetes care while also accommodating the harsh realities their patients face on a daily basis. Healthcare professionals should recognise the need to take their patients living situations and also any co-occurring conditions into consideration when they are developing their care plans. Having an integrated and co-ordinated model of care in specialised and social services have shown to improve the health outcomes in this vulnerable population (Baty et al. 2010). Additionally, tailored services specifically geared toward individuals who are classified as homeless have shown to improve the management of chronic disease such as diabetes (O’Toole et al. 2010).

This review also highlights some of the challenges the homeless population experience with managing their diabetes (Hwang and Chiu 2006; Hwang and Bugeja 2000). The findings have important implications to service provision and public health concerns given the prevalence and the significant morbidity of diabetes. Findings emphasise the importance of recognising that homeless status and the health of this population cannot be addressed in isolation. There needs to be action taken across the healthcare systems, for example, commissioning health services to undertake preventative measures.

The review has limitations due to the small number of studies currently available on the management of diabetes amongst the homeless population and the limited number of studies reporting on improvements of clinical outcomes as a primary outcome within their studies. The review may not be fully representative of the entire homeless population due to the low reported quality of the studies included, which was further demonstrated from the lack of details available on the participants characteristics. Therefore, future studies are needed to take into consideration cultural differences in defining and tackling homeless. In addition as most of the studies were from US and Canada, it would be essential to investigate in countries where the rates of homelessness and diabetes are high.

Findings from this review are consistent with previous studies that emphasise a need for management interventions amongst homeless individuals who have diabetes, which suggests that more quality data is needed (Gilani 2014; Jones and Gable 2014). However, despite the reported diabetes prevalence within this population, there are still significant gaps between the importance of managing the condition and the studies available to address those needs.

Future studies might wish to focus on exploring the health outcomes relating to the management of diabetes longitudinally and also assess the demographic and health indicators which are associated with participants concerns in relation to their diabetes condition. There is a need for comparative studies between the homeless and individuals who are housed in order to yield more epidemiologic data and also identify areas for further intervention. These will aid in investigating the barriers and challenges in managing the disease and accessing services for diabetes care. However priority should be given to the development of effective interventions and services in addressing the health needs vulnerable population groups. Studies focusing on diabetes interventions should explore the use of multidisciplinary treatments: the provision of basic necessities such as medication and healthy meals combined with outreach and educational programmes. Given the high burden of unmet medical and social needs, mobile interventions could prove to be particularly effective in this population group.

### Conclusion

Due to the chronic nature of diabetes, it is important that appropriate long-term support is available and accessible to individuals with diabetes. This synthesis of the available material focusing on diabetes interventions for homeless people highlights the need for a greater evidence base and high quality interventions to address health needs. Having targeted efforts in screening for the disease and support in managing the associated psychosocial factors of diabetes could aid in the improvement of health and social outcomes. A major challenge for healthcare providers is in providing high-quality diabetic care across all sections of society, which can prove to be more difficult when certain groups are hard to reach and engage with. Without appropriate support, such population groups are more likely to experience poor diabetes control and also be at a higher risk of developing major complications. Healthcare professionals and providers must recognise that this population group is a representation of those who are the most vulnerable and are more likely to benefit from effective interventions. However, the challenge lies in identifying, engaging and treating these individuals due to the scarcity of resources. Similarly, strong commitment and support from policymakers and politicians is vital for ensuring that the needs of this population are met. Successful approaches to diabetes management for this hard-to-reach population should be championed and learnings disseminated widely to promote replication in other areas and other population groups.
